# Notices and Cuttings

**Published:** 1858-08

**Authors:** 


### PRONOUNCING MEDICAL LEXICON.

*Pronouncing Medical Lexicon*, containing the correct pronunciation and definition of most of the terms used by speakers and writers on medicine and the collateral sciences, with Addenda; by C. H. Cleaveland, M. D., member of the American Medical Association: the American Association for the Advancement of Science; Prof. Materia Medica and Therapeutics in the Eclectic Medical College: second edition, Cincinnati, Longley Brothers, 1858.

This the only *Pronouncing* Medical Lexicon, has been long enough before the profession to have its reputation thoroughly established. The Dental Register of the West said of it: "This is a very convenient book for the student, and indeed, from its compactness and brevity, *exceedingly* useful to physicians."

As a *pocket* companion, or for ready reference, it has been prefered to all others, and its price being low, it is placed within the reach of those whose means are limited. It contains 300 pages, and is sold, postage paid, at $1,00. For sale by J. T. Toland.

"The Maxillatype Process for inserting artificial teeth, recently patented by Dr. Wm. M. Wright, has no similarity to any other plan now in use, to effect the same end, save in one particular,—the metal is poured in a melted state."

The above is copied from a circular, over the name of "Geo. W. Spencer, Agent for the sale of *Territory* and Office *Rights*. Patent issued, June 29, 1858. Office Rights, Fifty Dollars."

"If the Court understand herself, and she thinks she do," the above process is, in all material points, similar to Blandy's Cheoplasty process, the chief difference being in the composition of the metal; *Aluminium* and *Tin* being the principal components of the new candidate for favor. Whilst we have the best wishes for our friends, Dr. Wright and Dr. Spencer, we can not but predict for this, as for Cheoplasty, a *butterfly existence.*

It is to be regretted that restrictions are placed around every new improvement. The history of dental patents shows clearly that neither money nor reputation are to be made in that way, but both may be lost.

### NEW YORK DENTAL JOURNAL.

We have received the first No. of this new candidate for professional patronage. George H. Perine, Editor, Publisher and Proprietor. It takes an entirely new field in dental journalism. The classification is, 1st, Historical; 2d, Biographical; 3d, Bibliographical. The number before us is full of interesting and instructive matter, and from what we know of Dr. Perine personally, we are sure he will make each No. worth a year's subscription. He has our best wishes in his new enterprise. Published quarterly, at $2 per annum.

### COLLEGE JOURNAL OF MEDICAL SCIENCE.

We have had the pleasure of perusing several numbers of the above, and we must confess that we are surprised to see the amount of talent and valuable information displayed, in a *monthly* journal of its size, for only one dollar a year.

### DENTAL REGISTER OF THE WEST.

We have before us the June No. of this valuable adjunct to dental literature, filled, as usual, with interesting and useful information. Drs. Taft & Watt, the able and gentlemanly editors, are deserving of great praise for their energy and zeal in the cause of dental science. The present No. closes the 11th Volume, and we would suggest this as a good time to *subscribe*, beginning with the 1st No. of Vol. 12. The price is $3 a year, in advance, and we can safely say no one will regret the expense; for those who will read it carefully, and profit by what they read, will find *each No.* worth the year's subscription.

### DENTAL NEWS LETTER.

We are in receipt of the July No. of this sprightly and interesting publication. It comes, as usual, "chuck full" of valuable matter. It is so well known and so popular, it needs no commendation from us, but we would call especial attention of dentists, physicians and people, to an article on the use of prepared chalk and lime water, by J. D. White.

### AMERICAN DENTAL REVIEW.

This is a new quarterly, edited and published by our friend Dr. A. M. Leslie, of the St. Louis Dental Depot. As a proficient scholar, a bold and vigorous writer, and a perfect gentleman, we commend him and all his enterprises, to a liberal profession, feeling assured that they will reap an abundant harvest from their small investments.

### AMERICAN JOURNAL OF DENTAL SCIENCE.

This old and established Journal needs no commendation from us. It goes on in the even tenor of its way, Probably inclined a little to fogyism, it still exerts a wholesome influence, as a certain amount of conservative influence is needed in every society.

### WISCONSIN JOURNAL OF EDUCATION.

Published monthly at $1 a year, invariably in advance. We have received the June and August Nos. and can freely say we have met with nothing in this line, that so fully comes up to our views. We would be pleased to republish some of the articles, but want of space forbids at present. No man who feels an interest in the progress of Education should be without it. Address, "JOURNAL OF EDUCATION,"

*Madison, Wisconsin.*

### THE PRINTER.

A monthly newspaper, devoted to the "art preservative of all arts." Published by Henry & Huntington, New York.

This is an elegant work, full of illustrations and interesting matter, and should be in the hands of every printer. Price $1 a year in advance, or ten cents per number.

### WHO SPEAKS FIRST.

A gentleman with a good practice, Instruments, and office furniture nearly new, in a town of 7000 inhabitants, and only one other Dental office in the place, wishing to engage in another business, would dispose of his instruments, office and good will, on favorable terms, to one whom he could recommend to his patrons, this is a rare chance for any one wishing to locate in a growing place, with an established practice, for particulars, apply to

JNO. T. TOLAND,
DENTAL DEPOT, 38 *West Fourth street, Cincinnati, O.*

From the Cincinnati Daily and Weekly Times—so well known we need not commend it—its weekly circulation the largest in the West: devoted to the "American people:"

"*The Dental Reporter," for April.*—This is the first number of a new work just issued and edited by J. T. Toland, of this city, for the especial benefit of professional dentists. It is an independent journal, devoted to Dental progress and improvment in the manufacture and use of instruments and materials. The number before us contains some sixty pages, and is well filled with a most instructive and interesting variety of articles, among which are reports from several of the Dental Associations and Convention, with contributions from several gentlemen, well known to the profession generally.

The work is a quarterly, and is furnished at the price of twenty-five cents per year. In consideration of this very low rate it certainly can not fail to have a most extensive circulation. It is published by J. T. TOLAND, of No. 38 West Fourth street, Cincinnati.

---

From the Democratic Union, Somerset, Ohio, devoted to literature, art, science and politics. We can cheerfully recommend Barton Kagay, Editor, as a gentleman and a scholar:

*Dental Reporter.*—We have received the first number of the Dental Reporter, published quarterly, at Cincinnati, by John T. Toland, Editor, price 25 cents per year in advance, or 10 cents a number. It contains 58 pages, and will be devoted to the "production, cultivation, and preservation of good teeth." Mr. Toland says he has, within the past two years, printed and distributed among the dental profession 25,000 copies of his pamphlet, at a cost to himself of $2,500. Send us the Reporter Mr. Toland, you deserve well from the dental profession and the editorial fraternity should not forget you.

---

From the Wellsville Ohio Patriot, that sterling family paper, devoted to Literature, Science, Education, &c.

"The Dental Reporter and Independent Journal," devoted to dental progress and improvement, is the title of a very neat fifty page pamphlet, published quarterly by J. T. TOLAND, manufacturer of Dental instruments and wholesale dealer in dentists' materials, No. 38, West Fourth street, Cincinnati, now upon our table. The number before us contains several very important articles on the subject of dentistry, embracing almost every branch of the art, among which we observe a treatise on the science of excavation, filling and plugging, mounting teeth, rimming plates, extracting teeth, &c., &c.; and judging from the familiar manner with which each writer converses on his particular subject, we at once concluded that the best dental talent in the country is engaged on the work, and that it should be in the hands of every dentist in the State.

---

From the Newark Ohio Weekly Times, an excellent paper.

and the best advertising medium in that section. Edited by Mrs. C. P. Brister, a lady of talents and social worth.

We have received the first number of the Dental Reporter; an Independent Journal, devoted to Dental Progress and Improvements, in the manufacture and use of Instruments and materials. Edited by J. T. Toland, price 25 cents a year in advance; direct to the Editor, 38 West Fourth street, Cincinnati.

### POSTAL BON-MOT.

Dr. B. S. Codman, of the firm of Codman, & Shurtleff, Tremont Street, has handed us a letter-envelope in which was inclosed an articulated silver catheter, which came safely to hand under the guardianship of the following warning lines, addressed to the Post-office *employees.*

"In '*stamping*,' pray be quite discreet,
Though light, but frail and very neat,
Much force would *smash* it all complete!
Nor do into the contents peep;
It's but to '*tap*' the fountain deep,
From where so many troubles creep."

We do not wonder the little instrument arrived safe and sound, under such a touching and appropriate appeal.—*Boston Medical and Surgical Journal.*

### WESTERN DENTAL SOCIETY—THE SUPPER.

We have given, elsewhere, a brief notice of the proceedings of the annual meeting of the Western Dental Society, held in this city on Tuesday and Wednesday. This notice would be incomplete without a reference to the supper, given to the Society, at Lomelino's Hall, on Wednesday evening—a festive occasion given, we believe, by Drs. Lewis and Hubbard, of this city. Having the honor of an invitation, we had the pleasure of attending. It was one of the most pleasant affairs of the kind in which we ever participated. Wit and eloquence enlivened the occasion. Spirited and interesting speeches were made by Drs. Allport, Spaulding, Forbes, Branch, Lewis, Hubbard, McKellops, Barron, Peebles, Andrews, and others, and the toasts unusually pointed and piquant. We have rarely spent as pleasant an evening.

We can cordially assert that the Members of the Dental Society, present at this annual meeting, combine the elements of devoted attachment to their profession—high scientific attainments—and the attainments and requisites of social good-fellowship. Success to them! collectively and individually.—*Quincy Weekly Whig and Republican.*

From the Louisville Journal.

### CASE OF CHLOROFORM—HALLUCINATION IN LOUISVILLE.

Our readers no doubt remember the case of a dentist in Philadelphia who was accused and convicted of a rape upon a lady under the influence of chloroform, the lady herself being the only witness against him. There were many persons who doubted the reliability of the testimony of a person as to facts occurring during the influence of the chloroform, and it was a subject of much discussion. A very singular case has lately occurred in this city, showing how little such testimony is to be relied upon.

It seems that several of our most eminent physicians and surgeons, including Dr. Donne, Dr. S. Richardson, Dr. Cochrane, Dr. T. L. Caldwell, Dr. Colescott, Dr. Hardin, Dr. Bayless, and others, met to witness the removal by Dr. Goldsmith, the distinguished Professor of Surgery of the Kentucky School of Medicine, of a huge cancerous breast from the person of a lady residing in the lower part of the city. While an assistant was administering the chloroform, and before the patient was fully under its influence, she was observed to draw the covering over her breast, which was bared for the operation. Soon after this she sprang up and declared in the most indignant manner, that she "would rather die than be abused in that way." And it was only by the utmost efforts on the part of Dr. Goldsmith and the lady's husband that she could be induced to continue the use of the chloroform. After the operation was finished and the effects of the anesthetic had passed off, she was asked if she remembered anything of what had taken place. She answered, (her eyes, we are told, flashing with fury,) that she did not feel the cutting, but she knew well enough the indecent remarks made and the insulting liberties taken with her in her helpless state. She said that it was of no use to deny; that she had heard and felt all that had been said and done; and it was with difficulty she could be persuaded that her impression were a hallucination.

